# Relevance and application of digital project management in academic research: A case study in a Medical Physics lab and practice

**DOI:** 10.1002/acm2.70242

**Published:** 2025-09-01

**Authors:** Liesbeth Vancoillie, Milo Fryling, Ehsan Samei

**Affiliations:** ^1^ Center for Virtual Imaging Trials, Radiology Duke University Durham North Carolina USA

**Keywords:** collaboration, digital project management, research productivity, trainee development, workflow efficiency

## Abstract

**Background:**

The integration of project management (PM) methodologies in academic research is increasingly recognized as essential for improving research outcomes, enhancing team collaboration, and addressing issues of reproducibility and transparency.

**Purpose:**

This case study aims to evaluate the impact of implementing a digital project management platform in a medical physics research laboratory and affiliated clinical practice, focusing on improvements in project coordination, research productivity, and trainee development.

**Methods:**

A digital PM platform was introduced in a medical physics lab (CVIT) and clinical setting (CIPG) at Duke University to support coordination of research and clinical projects, streamline manuscript and conference submissions, and manage software tool and dataset development. Prior to implementation, project tracking relied heavily on email threads and spreadsheets, resulting in fragmented communication and delays. Usage data and qualitative feedback were collected over a 24‐month period to assess outcomes.

**Results:**

The implementation led to centralization of project information, more structured workflows, and real‐time collaboration. Improvements were observed in task delegation, progress monitoring, and team accountability. The number of actively managed projects increased, project completion rates improved, and manuscript submission became more efficient. Additionally, the platform fostered project management literacy among trainees, providing them with valuable organizational and communication skills viewed as formative for their careers.

**Conclusion:**

The integration of a digital project management platform improved research and clinical efficiency, transparency, and collaboration. It cultivated a culture of shared responsibility and structured progress tracking while supporting the professional development of trainees. These findings demonstrate the value of PM platforms in academic environments and provide practical insights for other research groups considering similar implementations.

## 1 INTRODUCTION

Project management (PM) methodologies and quality principles have long been standard in industry and business, contributing significantly to improved outcomes, streamlined workflows, and efficient use of resources.^1^ However, in academic and nonregulated scientific research, these approaches remain underutilized. Quality in research laboratories is often narrowly interpreted as accuracy, efficiency, data protection, and incremental improvements, while comprehensive quality management is still viewed by many as a resource‐intensive burden and a constraint on creativity.^2^ However, academic research continues to evolve into projects of bigger scopes with larger teams. At the same time, there is a growing recognition that the reliability and accuracy of scientific outcomes requires accountable, transparent, and reproducible processes. Persistent challenges of poor reproducibility and limited translation of research findings into real‐world applications highlight the urgent need for structured project management in academia. Traditional coordination tools such as email threads or shared spreadsheets can result in inefficiencies and communication gaps, especially as research becomes more interdisciplinary and complex. In this context, digital project management platforms offer centralized, dynamic environments for planning, delegation, communication, and resource allocation, supporting a culture of accountability and continuous improvement.^3^

According to the project management body of knowledge (PMBOK),^4^ a project is a “temporary structure established to create a unique product or service within certain time, cost, and quality constraints.” Academic research projects often meet this definition, as they pursue novel goals with dynamic teams and finite timelines. As such, research teams can benefit from modern project management tools just as much as, if not more than, traditional industries. One such tool is Monday.com, a cloud‐based project management platform designed to facilitate collaboration, track progress, and manage tasks through customizable workflows.^3^ Athough Monday.com is widely used in the corporate sector due to its user‐friendly interface and high performance across multiple evaluation criteria, its application in academic research remains limited. There is a need and opportunity to explore the potential of this digital management method to increase transparency, streamline communication, and improve coordination in a research setting.^5^

The center for virtual imaging trials (CVIT) and the clinical imaging physics group (CIPG) at Duke University implemented Monday.com to address its need for project management. In particular, the goal was to assess how this tool might help with challenges such as unclear task delegation, delays in manuscript submissions, and lack of oversight in software and dataset development. This case study examines how the platform supported project planning and execution in three key domains: (1) coordination of research projects, (2) tracking of manuscript and conference submissions, and (3) development of software tools and datasets. We evaluate the impact of the platform on efficiency, collaboration, and accountability, and provide practical guidance for other academic or clinical groups considering similar implementations.

## 2 MATERIALS AND METHODS

### 2.1 Research environment and project structure

Our CVIT research lab operates in a dynamic, interdisciplinary setting, with most projects led by doctoral or master's students. The CIPG practice consists of medical physicists who lead projects primarily initiated based on the hospital's needs. These lead individuals coordinate with collaborators from medical physics, radiology, computer science, and engineering. Because research projects often involve simultaneous contributions from multiple stakeholders, transparent communication and well‐structured workflows are essential for maintaining progress and consistency.

To support this, we selected Monday.com, a cloud‐based project management platform, based on its user‐friendly interface, flexible template system, and integration with existing tools. Our evaluation of several tools (including Monday.com, Asana, and clickUp) highlighted Monday.com's ability to balance customization and standardization, which was critical for our highly variable research environment.

Prior to its adoption, project tracking relied on email chains, spreadsheets, and in‐person updates, which frequently led to delays. The new system enabled real‐time visibility across all stages of project development, improved traceability, and enhanced coordination across team members. Key implementation milestones included migration of existing projects, training workshops, standard operating procedure (SOP) development, and the creation of customized boards for onboarding, project tracking, and data curation. After several months of use, these boards were refined to enhance user engagement; unnecessary elements were removed, and missing features were added based on observed needs and user feedback.

### 2.2 Member ownership and involvement

All CVIT and CIPG members with temporary appointments for longer than 6 months have been granted full access to the digital management system. Architectural and system‐level changes remain restricted to the three designated administrative leaders. We developed a standardized process to streamline the onboarding of new members. The person responsible for the new addition, whether an undergraduate student, graduate student, PhD student, visitor, postdoctoral researcher, employee, medical physicist or medical student, initiates this process using a SOP template. This template collects key information, including the Duke NetID, administrative needs such as access to the Portal, Slack, badge, and mailing list, IT needs such as access to the cluster, server, VPN, and Docker, training requirements such as GitLab or Monday.com training, the start and end dates, and the name of the person who requested the onboarding. All entries are tracked in the corresponding Onboarding Board on Monday.com. This ensures a consistent and transparent start for each new team member and represents the first step in maintaining a comprehensive overview of everyone working in or joining the lab.

### 2.3 Project definition

At the core of the system was the concept of a project, defined as a prospectively planned, interdependent effort typically requiring 2 to 6 months to complete. Each project was succinctly described using the key elements listed in Table 1. This structured approach promotes intentional, critical thinking and helps ensure a shared understanding among all involved. To ensure intra‐lab reproducibility and streamline onboarding, we implemented a lab‐wide SOP policy. A dedicated SOP template, integrated into Monday.com, guides project leads through the structured planning process before project initiation. By requiring accountability and clearly defining standards for data and process use, SOPs improve reproducibility and institutional memory, especially when projects lead to publications or collaborations.

**TABLE 1:** Overview of project documentation elements, including definitions and illustrative examples used for consistent characterization across initiatives.

Element	Definition	Example
Project Number	Unique identifier assigned by the ‘XX’ and ‘XX’ project manager	53
Title	Concise project title	Modeling lung respiration motion
Lead	Primary person responsible for the project	Jane Doe
Team	Proposed team members, each with a defined role	Morgan Blake, Dr. Sam Sample, Casey Morgan
Supervisor	Project overseer	Dr. John Smith
Gap	Specific need or problem the project aims to address	Lung CT scans may be acquired at inadequate lung ventilation or during respiration. Current lung respiration model is based on average 4D CT motion trends, which lacks accuracy and variation due to disease.
Objective	Clear goal or aim of the project	To develop an accurate respiration model that includes disease and patient specific diaphragm and rib motions/forces.
Actions	Succinct list of planned actions	‐Conduct literature review to identify approaches ‐Select and implement method ‐Integrate disease‐specific features ‐Generate phantom models at different respiration levels ‐Evaluate and refine the model
Outcome	Expected deliverables	‐Respiration model framework (software) ‐Lung models at different respiration levels (data) ‐Journal paper ‐Conference presentation
Start date	Project start date	Jan 23, 2024
Status	Current state	Parked, Underway, Completed, Terminated, Future
Health	Project condition	On track, Completed, At risk, Stuck, Terminated
Parent Project	Prior project from which this project originated, if applicable	12

### 2.4 Project lifecycle and workflow

**FIGURE 1:**
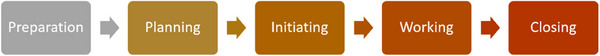
The five stages of the project lifecycle.

We implemented a structured yet adaptable project workflow that mirrors the project management lifecycle and encourages student leadership, collaboration, and accountability (see Figure 1).^5^ The following phases outline the typical trajectory of a project within our lab.

**Preparation phase**: In this early phase, members review the theoretical underpinnings of project development and discuss project ideas with their supervisors (often the advisors in the context of student trainees). Group meetings are held to align ideas with practical needs and determine gaps the project could address. Members are encouraged to reflect on potential deliverables and assess whether the project aligns with lab objectives and external grant requirements.

**Planning phase**: Once a concept is selected, the project lead completes a standardized project intake form via Monday.com. This form prompts the lead to articulate the project's elements (Table 1). The CVIT and CIPG project manager then reviews the submitted proposal, sets up the corresponding project board, and assigns a standardized identifier to ensure traceability and avoid redundant efforts. Although this structured intake sets the foundation, flexibility remains a core principle, allowing leads to adapt as their projects evolve.

**Initiation phase**: Upon approval, the project is formally launched in Monday.com. Leads finalize team roles and responsibilities, define deliverables, and draft a work plan using actionable items. They also outline the required methods and identify whether resources beyond those of the lab will be needed.

**Working phase**: This is the most intensive part of the project lifecycle. Project leads and their teams execute their plans, often iterating between group and individual tasks. Regular targeted meetings every 2 weeks offer a platform for discussion and peer learning across projects with similar themes. Collaboration with external partners is supported where relevant. Supervisors provide feedback, motivation, and strategic input throughout this stage, helping trainees navigate challenges and stay aligned with deliverables.

**Closing phase**: A project can only be considered complete if at least one of four core deliverables (paper, abstract, dataset, or software) has been produced. Upon completion, the lead presents the results to the supervisors and the project manager. At this stage, deliverables are verified: software is reviewed by the IT team for integration into the lab's GitLab repository, and datasets are checked for accessibility and proper documentation. The final review also includes an evaluation of manuscripts and abstracts.

### 2.5 Platform implementation

To support efficient workflow management for the various projects in our lab and clinical practice, we developed customized spaces in Monday.com, each designed to serve a specific function within our research pipeline. These boards help streamline task delegation, track progress across different phases, and ensure clear communication among team members, ultimately contributing to more organized and productive project execution. The boards are collected in the general CVIT or CIPG Portfolio.

**FIGURE 2:**
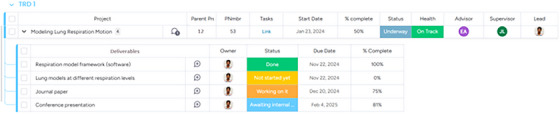
Example of a personalized active project view for a CVIT or CIPG member. Each active project displays the parent number, project number, a link to the project workspace, start date, percent complete, status, project health, and assigned members. Within each project, users can view associated deliverables, including their owner, status, due date, and percent complete.

**Active project board**: The dedicated active project board centralizes project activities and milestones. It provides a comprehensive overview of task lists, deadlines, and dependencies, ensuring that project phases and milestones are completed in a timely manner (see Figure 2). This board is structured into several key sections, including a project overview that offers a high‐level summary of each project, detailing its objectives, key personnel, and expected deliverables. Task management involves detailed tracking of individual tasks, assigned team members, priority levels, and due dates. Progress monitoring is supported through a real‐time status update system that categorizes tasks such as “Not started yet,” “Working on it,” or “Done.” Additionally, resource allocation is managed through a dedicated space for tracking personnel assignments and necessary resources. The active project board facilitates structured project execution, enabling efficient resource allocation and real‐time progress tracking, thereby minimizing bottlenecks in research workflows.

**Project workspace**: Each active project listed in the active project board is linked to its own dedicated project workspace. These workspaces are managed by the project leads and team members, and are designed to be flexible and adaptable to the specific needs and working styles of each project. Individual workspaces include clearly defined actions, responsible team members, and customizable columns that allow for tailored tracking of priorities, deadlines, and specific workflows. In addition to task management, these workspaces are also used for purposes such as bug tracking and documentation updates. By enabling focused, project‐specific coordination, the individual workspace complements the centralized system and contribute to efficient day‐to‐day project execution.

**FIGURE 3:**
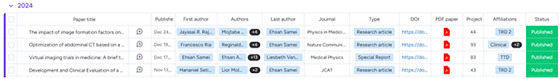
Example view of the paper board used in the CVIT and CIPG portfolios. Each entry includes fields for published date, authors, journal, type, DOI, PDF link, project number, affiliations, and status.

**Paper board and abstract board**: To manage academic papers associated with our research, the paper board tracks the status of each manuscript from preparation through internal review, submission, acceptance, and publication (see Figure 3). This system facilitates real‐time monitoring, ensuring that papers progress through the review and publication stages efficiently. The board includes a manuscript tracker that organizes all ongoing and past manuscripts, listing key authors, journals, deadlines, and submission statuses. Review status displays reviewer feedback, required revisions, and final acceptance timelines. On the abstract board, conference abstracts are monitored, with tracking of abstract submissions, deadlines, acceptance notifications, and presentation details. In addition, collaboration tools enable co‐authors to share drafts, comment on sections, and update each other on progress. This structured approach ensures that publication timelines are adhered to, reducing delays and improving the efficiency of academic dissemination.

**FIGURE 4:**
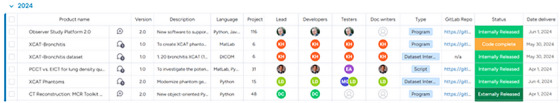
Example of the software and dataset board for the CVIT lab in 2024. Each entry includes the product name, version, description, language or format, project number, lead, developers, testers, documentation writers, product type, GitLab repository, product status, and delivery date.

**Software and dataset board**: For projects involving software development or (virtual) dataset creation, the software and dataset board manages coding activities, release timelines, and GitLab repositories. This structured approach promotes collaboration and ensures that development milestones are achieved in an organized and timely manner. The board tracks major milestones in software and dataset creation, including a version number, coding phases, testing, validation, and release dates (see Figure 4). Bug tracking is incorporated to log identified issues, assigned developers, and resolution statuses. Version control is integrated with GitLab to monitor software versions, repository changes, and feature updates. Additionally, data management tools organize datasets, metadata, and access permissions to ensure proper documentation and reproducibility. The board also keeps track of what has been completed, internally or externally released, along with the corresponding release dates. This centralized system streamlines the research development cycle, enhances efficiency, and minimizes errors.

**FIGURE 5:**
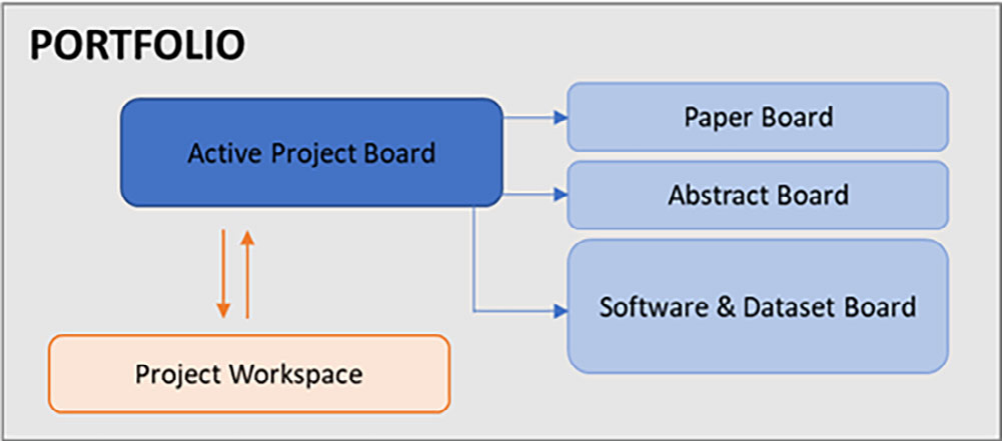
Relationship between the active project board, paper board, abstract board, software and dataset board, and the associated project workspaces, which are customized Monday.com boards used in the CVIT and CIPG portfolios.

For the CVIT and CIPG portfolio, these are the most important boards for the project management part of the center. Figure 5 shows the relationship between these customized Monday.com boards. In addition to the boards dedicated to tracking the progress of individual projects, we have also developed supplementary boards aimed at maintaining a high‐level overview of lab activities. These boards consolidate information across projects, highlight key milestones and deadlines, and provide a centralized space for team coordination, resource planning, and strategic decision‐making.

**FIGURE 6:**
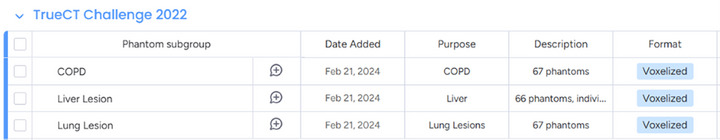
Phantom group 'TrueCT Challenge 2022′ with information about the date added, purpose, description, and format included.

**Phantom database board**: We created a dedicated board to serve as a centralized database for all the anthropomorphic phantoms (XCAT) developed in the CVIT lab. The board includes a summary page organized by phantom group, with each group representing a major version of the XCAT phantom. Figure 6 presents an example of the group ‘TrueCT Challenge 2022′. For each phantom group, we document its subgroups, where we record the date each was added, its intended purpose, the number of phantoms it contains, and the file format. Each phantom group links to a more detailed section listing the individual phantoms within its subgroups. For every phantom, we record key metadata as outlined in Table 2. This structured database allows us to keep track of all phantom development within the lab. It enables trainees and researchers to easily explore available phantoms, export relevant information, locate associated files on the server, and incorporate them into their own studies.

**TABLE 2:** Metadata elements associated with each phantom in defined groups, detailing input parameters, anatomical and demographic characteristics, and simulation properties.

Element	Definition
Source ID	Unique identifier of the base subject
File name	Name of the file stored on the server
Input parameters	Parameters used to generate the phantom in the simulation platform
Organ materials	Material file assigned to the phantom
Anatomical texture	Whether texture was included
Body region coverage	Anatomical region(s) covered
Motion status	Whether the phantom includes motion simulation or not
CT Image origin	Original CT scan source (e.g., institution or dataset)
Sex	Biological sex of the phantom subject
Age	Age in years
Weight	Weight in kilograms
Height	Height in centimeters
BMI	Body mass index
Race	Reported race or ethnicity

**Invited talks board**: We maintain a dedicated board to track invited talks delivered by members of the lab. This board records details such as the speaker's name, title of the talk, event or institution, date, and relevant materials or links. It serves as a centralized record of our lab's external engagement and scientific visibility, and helps ensure that contributions are documented and recognized across the team.

**Collaboration board**: We engage in research collaborations with institutions beyond our own. To manage and document these activities, we maintain a dedicated board that tracks key information for each collaborative project. This includes the research focus, principal investigator, institution, funding source, country, start date, and project status (active, paused, terminated, or planned for the future). The board also identifies the lab member responsible for coordinating each collaboration. This centralized system supports internal oversight and facilitates the preparation of our annual progress report by providing a clear overview of all ongoing and completed collaborative efforts, along with the contributions of our partners.

### 2.6 Feedback and communication

In addition to continuous monitoring via Monday.com, several feedback mechanisms are in place to support communication and ensure project alignment. Biannual Monday reports serve as a structured tool to evaluate project progress and provide targeted feedback to team members. Regular check‐ins with the lab manager offer an opportunity for ongoing support and timely adjustments, helping trainees stay on track and address challenges as they arise. Additionally, annual orientation sessions are organized to introduce new lab members to the project management system and outline the lab's expectations, fostering a shared understanding of workflows and responsibilities from the outset.

### 2.7 Project management framework and skill development

Beyond workflow optimization, our framework aimed to build essential project management skills in trainees. Drawing from the literature on PM education, three core competencies were emphasized: (1) critical thinking for navigating complex projects, (2) integration of interpersonal and technical skills, and (3) preparation for leadership roles in varied contexts [5, 7, 8]. Our system fostered responsibility, collaboration, and communication, equipping trainees with 21st‐century skills valued by employers. The project intake process required early reflection on project goals, structure, and gaps, while SOPs and regular updates encouraged accountability and attention to detail. By combining structured tools with educational intent, the framework contributed both to lab efficiency and to trainee development beyond academia.

### 2.8 Evaluation of impact

To assess the impact of Monday.com on research coordination and productivity, we conducted a 24‐month evaluation (January 2023 to December 2024), using a mixed‐methods approach. Evaluation metrics were selected to reflect improvements in project coordination, research output, documentation practices, and professional development. These included both quantitative indicators and qualitative feedback. Quantitative data were gathered from system usage analytics, such as the number of active projects, board activity frequency, and project durations, as well as from project lifecycle logs tracking parked versus completed projects, submission timelines for abstracts and manuscripts, and records of publications and software or dataset releases. Qualitative data were obtained through biannual structured interviews with trainees and supervisors, and performance review discussions assessing growth in project management skills. These methods allowed us to triangulate evidence from multiple sources, offering a comprehensive understanding of the platform's effect on lab operations. Metrics such as increased project visibility, reduced delays, earlier supervisor involvement in writing, and improved standardization of deliverables were used to evaluate the effectiveness of the system. Importantly, the data collection was embedded in the regular project management cycle, enabling continuous monitoring and course correction without imposing additional administrative burden.

## 3 RESULTS

The 24‐month mixed‐methods evaluation yielded clear evidence of improved research coordination, output, documentation, and professional development following the integration of Monday.com. The intuitive interface and customizable features of the platform accommodate diverse technical backgrounds, allowing all team members to engage effectively in project activities. In the sections below, we present quantitative metrics and qualitative insights structured according to the four primary evaluation domains: (1) project coordination and transparency, (2) research output and submission practices, (3) documentation and reproducibility, and (4) trainee development.

### 3.1 Enhanced project coordination and transparency

Following the implementation, the number of actively managed projects increased from 58 to 73, without any increase in management overhead. Meanwhile, the number of parked projects decreased from 17 to 7, as closer monitoring allowed us to identify and address the underlying reasons for delays. The number of completed projects remained consistent across half‐yearly periods, with 4, 8, 12, and 9 completions respectively, indicating a stable project completion rate. The introduction of project workspaces enhanced team alignment by improving delegation and clarifying responsibilities. We also began systematically tracking the duration of ongoing projects, with a focus on those exceeding 1 or 2 years. This close monitoring helped prevent projects from becoming significantly delayed: only 14 out of 24 projects exceeded the 2‐year mark after passing the 1‐year threshold. Given that our ideal project timeline ranges from 3 to 9 months, depending on the nature of the task and whether it involves a master's or PhD student, we aim to minimize the number of long‐running projects. The proportion of projects exceeding the target duration decreased from 39% in 2023 to 26% in 2024, reflecting improved timeline adherence. Additionally, the use of Monday.com for regular updates ensures full transparency into ongoing activities. Every CVIT and CIPG member has easy access to relevant information, making it easier to identify overlapping research and collaborate effectively.

### 3.2 Increased research output and timely submissions

The implementation of the structured paper board and abstract board contributed to a 65% increase in accepted abstracts, from 23 in 2023 to 38 in 2024. Although the number of published papers remained relatively stable (21 in 2023 and 18 in 2024), the overall research output was maintained at a high level. A notable shift was observed in the timing of feedback requests: supervisors were consulted earlier in the writing process, which facilitated more constructive input and improved final submissions. Board activity logs showed an increase in tasks tagged for ‘Internal Review’ within the early stages of manuscript development, allowing the research manager to follow up proactively and facilitating earlier journal submissions. Trainees reported a greater sense of control over their timelines, while supervisors appreciated the improved visibility into project status, enabling them to provide timely and targeted guidance.

### 3.3 Improved documentation and reproducibility

The implementation of the software and dataset board significantly improved standardization across development pipelines. In 2023, of the 10 software tools completed, one was released externally and two internally. In 2024, this increased to two external and three internal releases. All remaining completed tools were properly tracked and made accessible, as each entry on the board was linked to a dedicated GitLab repository. Importantly, all software and datasets released during the evaluation period included complete metadata documentation and version control logs, elements that were not consistently controlled or verified prior to the introduction of this board. Additionally, all datasets released (two external, four internal) adhered to the internal SOP format and were archived with standardized metadata. This improved both the transparency of the development process and the potential for reuse in future projects.

### 3.4 Development of key project management competencies

Qualitative feedback and performance reviews highlighted notable growth in trainee leadership, planning, and communication skills. Trainees reported greater clarity about project goals and expectations. Supervisors observed improved preparation in project presentations and more proactive problem‐solving. A follow‐up survey indicated that trainees felt more confident managing complex projects after using the platform, and stated the experience improved their readiness for professional environments beyond academia. Trainees agreed or strongly agreed that Monday.com helped them develop skills relevant to future careers, and they reported greater confidence in leading or coordinating research tasks.

The board templates developed in our lab can be shared with the broader research community, fostering standardized and efficient project management practices beyond our institution.

## 4 DISCUSSION

The adoption of Monday.com in our academic lab addressed several challenges related to communication, accountability, and project coordination. The platform significantly improved the management of active projects, reducing stalled initiatives and ensuring quicker interventions when issues arose. This aligns with previous findings showing that early detection of project bottlenecks is critical to maintaining research momentum in academic environments.^6^ By structuring workflows, the digital management system helped maintain consistent research output and fostered greater team engagement, demonstrating that project management tools can enhance academic research without hindering creativity or autonomy.

A key strength of Monday.com lies in its adaptability. The customizable boards and standardized templates allowed us to tailor the platform to various project types and team dynamics while promoting consistency in execution and documentation. The use of an intake form encouraged early strategic planning and clarified expectations, while regular check‐ins and reports ensured alignment with lab goals and provided a feedback loop for continual progress. The increase in early feedback requests suggests that earlier supervisor engagement, enabled by these structured workflows, contributed to improved writing quality and reduced last‐minute submissions.

Our results also point to tangible gains in research output and reproducibility practices. For instance, the paper and abstract boards corresponded with an increase in accepted abstracts and a notable improvement in feedback timing. Meanwhile, all externally released datasets and tools adhered to internal SOPs and included full metadata, underscoring improved adherence to documentation standards.

In addition to project management skills, the members strengthened their essential qualitative behaviors through working with project management platforms. They gained an understanding of the broader context by researching the field of their project application, fostering the ability to “see the bigger picture.” Leadership skills were cultivated as members reflected on key moments in their projects, such as motivating the team and overcoming challenges.^5^ Team dynamics and individual personalities were professionally mitigated as individuals navigated their collaborative work, learning how to share responsibilities, communicate effectively, and reach consensus. Decision‐making was emphasized as trainees realized that every choice impacts the outcome of the project. These outcomes, supported by structured interview data and consistent performance reviews, suggest that platforms like Monday.com can serve not only as organizational tools but also as professional development environments. These experiences prepare trainees for real‐world project management challenges while enhancing their ability to work effectively within diverse teams.

The success of the platform depends on sustained user engagement and timely updates, which require ongoing oversight. Not all team members adopted the system with equal enthusiasm, underscoring the need for cultural change and additional training. Although automation and structured workflows improved many operational aspects, they cannot replace the importance of interpersonal communication and mentorship. Future studies could assess the long‐term retention of these skills and explore comparative outcomes across teams using different management systems, thereby expanding the evidence base on the role of digital tools in academic research productivity.

## 5 CONCLUSION

This case study demonstrates how academic labs and clinical practice can benefit from modern project management tools like Monday.com. By enhancing clarity, coordination, and accountability, the platform improved project execution and supported skill development among trainees. Although challenges in adoption and engagement remain, our experience highlights that structured project management systems can significantly enhance the quality and efficiency of academic research, particularly in interdisciplinary and data‐driven settings. Our use of digital project management notably increased the efficiency and effectiveness of our research environment and progress, showcasing the value of digital platforms in streamlining operations, fostering collaboration, and maximizing outcomes. The insights from this experience offer a model for other academic institutions aiming to improve research efficiency and accountability.

## AUTHOR CONTRIBUTIONS

Liesbeth Vancoillie conceptualized the study, conducted the analysis, and drafted the manuscript. Milo Fryling contributed to the conceptualization of the use of the project management platform implementation, follow up, and manuscript revision. Ehsan Samei provided supervision and critical feedback. All authors reviewed and approved the manuscript.

## CONFLICT OF INTEREST STATEMENT

The authors declare no confict of interest.
